# *Glycyrrhiza* Polysaccharide Alleviates Dextran Sulfate Sodium-Induced Ulcerative Colitis in Mice

**DOI:** 10.1155/2022/1345852

**Published:** 2022-04-08

**Authors:** Chunying Huang, Xiaoqi Luo, Lulu Li, Nan Xue, Yuanjie Dang, Hongli Zhang, Jingxuan Liu, Jibing Li, Cuiqin Li, Farong Li

**Affiliations:** ^1^Key Laboratory of Medicinal Resources and Natural Pharmaceutical Chemistry & Ministry of Education & National Engineering Laboratory for Resource Developing of Endangered Chinese Crude Drugs in Northwest of China, College of Life Sciences, Shaanxi Normal University, Xian, Shaanxi Province 710062, China; ^2^School of Basic Medical Science, Chengdu University of Traditional Chinese Medicine, Chengdu, Sichuan Province 611137, China

## Abstract

**Background:**

Licorice is one of the most ubiquitous herbs in traditional Chinese medicine, with notable anti-inflammatory and antiulcerative effects as well as potent digestive disease therapeutic impacts; yet, its active components and mechanisms remain unclear. There is a lot of evidence that *Glycyrrhiza* polysaccharide (GPS) has antioxidants, improving intestinal flora, anti-inflammatory effects, etc. *Hypothesis/Purpose*. Here, we investigated the effects of GPS on dextran sulfate sodium (DSS)-induced acute ulcerative colitis (UC) mice and its possible mechanisms.

**Methods:**

GPS (100, 200, and 400 mg/kg) or the positive control drug sulfasalazine (SASP) (200 mg/kg) were orally administered to mice for 8 days. Body weight was recorded daily. Symptoms associated with UC, such as disease activity index (DAI), colon length, spleen weight, and mucosal damage were detected. The possible mechanism of GPS ameliorating enteritis symptoms was explored by detecting intestinal permeability and serum levels of inflammatory factors, and changes in intestinal permeability were expressed by serum concentration of FITC-dextran and D-lactic acid.

**Results:**

The results demonstrated that GPS administration alleviated UC symptoms in colitis mice, including weight loss, DAI index, shorting colon length, and mucosal damage. Mechanistic evaluation revealed that GPS treatment reduced intestinal permeability and serum levels of inflammatory factors: IL-1, IL-6, and TNF-*α*, while increasing serum levels of the anti-inflammatory factor IL-10, suggesting that GPS's mechanism in UC is related to reducing intestinal permeability and inhibiting the inflammatory response, with intestinal permeability implicated as the initiating mechanism.

**Conclusion:**

This study highlights GPS as a promising therapeutic agent, with high therapeutic efficacy and a good safety profile, for enteritis and beyond.

## 1. Introduction

Ulcerative colitis (UC) is a recurrent chronic gastrointestinal disease that seriously endangers human health [1]. UC lesions typically are present in the colonic mucosa and submucosa; its clinical symptoms include weight loss, bloody diarrhea, a potential increase in colon cancer risk, and in severe cases death [2]. UC is predominantly characterized by increased intestinal permeability and inflammation responses, and restoring mucosal barrier function has been theorized as a possible therapeutic approach to combat this disease [3]. Numerous factors are implicated in UC pathogenesis, including the deletion and variation of a series of susceptibility genes, environmental changes, abnormalities of the intestinal microbiota, and widespread immune dysregulation; however, the specific pathogenesis of UC remains unclear [4]. Current UC therapeutics target suppressing immune reactions and reducing inflammatory factors, with the short-term treatment goal of reducing mucosal inflammation and mucosal damage [5]. Notably, these drugs have severe adverse effects such as vomiting, anemia, and generalized edema [6]. Moreover, while minimizing mucosal inflammation and damage are admirable short-term goals, mucosal healing could provide similar immediate benefits as well as achieve longer-term treatment outcomes [7].

Licorice is widely used across Chinese medicine formulas for its anti-inflammatory, antiulcer, heat-clearing, and detoxifying effects, with it said that “9 out of 10 formulas contain licorice” [8]. According to the Chinese Pharmacopoeia, licorice refers to the roots of *Glycyrrhiza uralensis* Fisch., *Glycyrrhiza inflata* Bat., or *Glycyrrhiza glabra* L. used as a medicinal material (State Pharmacopoeia Committee, China, 2020). Research has shown that licorice extracts exhibits a good therapeutic effect on ulcerative colitis [9], with numerous potential active ingredients identified: polysaccharides, saponins, and flavonoids [8].

In particular, studies have shown that components in licorice have excellent anti-inflammatory properties [10]. Glycyrrhizin has been shown to reduce levels of nitric oxide, TNF-*α*, and PGE2 in LPS-stimulated macrophages. Glycyrrhetinic acid and glycyrrhizin have also exhibited anti-inflammatory effects via the inhibition of proinflammatory cytokines IL-6 and IL-1*β*, and regulation of the NF-*κ*B pathway in LPS-treated RAW264.7 macrophages [11], Furthermore, glycyrrhizic acid has been considered one of the major active components in the treatment of ulcerative colitis [12]. Glycyrrhiza polysaccharide (GPS) has recently been implicated as another primary active ingredient in licorice, with studies finding that GPS can regulate the immune system and gut microbial ecosystem [13]. Notably, gut microbiota imbalance is a main feature of UC, and several studies have confirmed that fecal microbiota transplantation could improve UC symptoms [14]. In a previous study, our group coincidentally found that GPS could dramatically increase tight junction protein ZO-1 expression in immunosuppressed mice intestinal tissues (Figure S1). Given that damage to the cell tight junction may cause an increase in intestinal permeability, a typical characteristic of UC, and GPS's effect on the immune system and gut microbial ecosystem, we hypothesized that GPS might ameliorate UC symptoms.

Herein, we quantify GPS's effect of on DSS-induced acute UC mice as well as its effects of on intestinal permeability and inflammatory factors. This experiment may deepen the understanding of traditional licorice use and illuminate licorice's active substance for further therapeutic utilization.

## 2. Material and Methods

### 2.1. Animals

Sixty male Kunming mice, weighing 18 ± 2 g, were purchased from the Laboratory Animal Center of the Air Force Medical University of PLA (Xi'an, Shaanxi, China) with an animal production license number of SYXK (Shaanxi) 2014-001. All mice were bred in a standardized animal room, with free water and food intake, maintained indoor ventilation conditions, and normal day and night cycles. Relative humidity was maintained between 40 and 70% and room temperature between 18 and 22°C. Animals were acclimatized for 7 days. The experimental protocol was approved by the Committee on the Ethics of Animal Experiments of Shaanxi Normal University (no. ECES-2018-0223).

### 2.2. *Glycyrrhiza* Polysaccharide (GPS) Preparation

Radix Glycyrrhiza was purchased from Tianjin China Medico Technology Co., Ltd, and was identified as the root of *Glycyrrhiza uralensis* Fisch by professor Lijuan Zhang. The voucher specimens were kept by Tianjin China Medico Technology Co. Ltd., specimen number SSBC-G-055. 1 kg of dried Radix Glycyrrhiza was milled with a multifunctional grinder, diluted with distilled water to a 1 : 10 (w/v) ratio, and then extracted for 2 h at 90°C; this process was repeated two additional times. Subsequently, the extract was concentrated and settled overnight in 80% ethanol. The precipitate was then collected by centrifugation. After protein removal via the Sevag method, the solution was concentrated and lyophilized to obtain crude *Glycyrrhiza* polysaccharide (GPS). The yield of GPS was calculated by following equation:

Yield (w/w%) = M1/M2 × 100% (M1: weight of dried GPS; M2: weight of dried Radix Glycyrrhiza).

### 2.3. GPS Molecular Weight Determination

The average GPS molecular weight was determined by gel permeation chromatography: Agilent 1200 HPLC system (Agilent Technologies Co., Ltd., USA); column, Dr. Maisch GembH Reprosil 125 SEC; oven temperature, 30°C; mobile phase, deionized water; flow rate, 0.5 mL/min; ELSD detector. Molecular weight standard curves were obtained via dextran standards of differing molecular weights (1.98 × 10^2^, 4.32 × 10^3^, 1.26 × 10^4^, 7.08 × 10^4^, 1.26 × 10^5^ g/mol); the molecular weight of GPS and its fractions were calculated then by the standard curve.

### 2.4. GPS Monosaccharide Composition

GPS was hydrolyzed in 2 mol/L trifluoroacetic acid solution, and the composition of the hydrolyzed products analyzed by HPLC after precolumn derivatizated with 1-phenyl-3-methyl-5-pyrazolon (PMP) (Solarbio Life Science Co., Ltd, Beijing, China) [15, 16]. Monosaccharides were analyzed via an Agilent 1200 HPLC system (Agilent Technologies, Santa Clara, CA, USA) with a quaternary pump, autosampler, degasser, and automatic thermostatic column compartment. A ZORBAXSB-C18 (4.6 mm × 150 mm, 5.0 *μ*m) column was used. Monosaccharide standards: D-glucose (Glu), D-mannose (Man), L-rhamnose (Rha), D-glucuronic acid (GlcUA), D-galactose(Gal), D-galacturonic acid (GalUA), L-arabinose (Ara), and D-xylose (Xyl) were purchased from the National Institute for Food and Drug Control (Beijing, China).

### 2.5. DSS-Induced UC Mice Model and Animal Treatment

After the 7 day adaptation period, mice were randomly divided into 6 groups according to body weight. The 6 groups were the normal control group (NC), model group (Model), high-dose GPS group (GPSH, 400 mg/kg·d), medium-dose GPS group (GPSM, 200 mg/kg·d), low-dose GPS group (GPSL, 100 mg/kg·d), and positive drug group administered with SASP (SASP, 200 mg/kg·d) (Sinopharm Chemical Reagent Co., Ltd, Shanghai, China); 10 mice per group. The UC mouse model was induced by 5% (w/v) dextran sulfate sodium (DSS, 36000-50,000. Mw) (Santa Ana, California, USA) in the mice drinking water for 6 days [17]; the normal control group drank water without DSS. After modeling, all 6 mice groups drank purified water. GPS and SASP were administered intragastrically, respectively, from day 7 to day 14. Meanwhile, mice in the NC and Model groups received the same volume of vehicles solvent. On day 15, all mice were anesthetized and orbital blood was collected, spleens were dissected and weighed, and the entire colons were dissected and their lengths measured. Sections of rectums were taken and fixed in 10% neutral buffered formalin for subsequent pathological analysis.

### 2.6. Evaluation of UC Progression

Disease activity index (DAI) scores, spleen index, and colon length were used to evaluate UC progression. DAI scores were based on weight loss, fecal structure, and fecal occult blood [18], and the DAI scoring criteria are shown in Table 1.

### 2.7. Histological Examination

Histopathological studies were performed on paraffin embedded, 5 *μ*m thick distal colon sections, and stained with haematoxylin and eosin, followed by a blinded scoring of histologic lesions as described previously in the literature [19].

### 2.8. FITC-Dextran Serum Content

FITC-dextran (FD4) serum concentration was used to assess mice intestinal permeability. All mice were fasted for 4 h, then intragastrically administered 0.2 mL FITC-dextran solution (FITC-dextran, 4 kDa, 40 mg/100 g) (Sigma-Aldrich, USA). All mice were then rested for 5 h without food or water. After anesthesia, blood was collected from orbital sinuses. The blood was then placed into 1.5 mL conical tubes and centrifuged at 3500 rpm for 10 min at 4°C. 75 *μ*L of each serum, in triplicate, were then aliquoted into a 96-well plate. FITC-dextran fluorescence was then measured on a fluorescence plate reader via suitable wavelengths (excitation maximum 488 nm, emission maximum 525 nm) [20]. The exact FITC-dextran serum concentration was then calculated according to FITC-dextran standard curves.

### 2.9. D-Lactic Acid Serum Content

ELISA was used to detect serum D-lactic acid content according to the manufacturer's instructions on the kit. A linear regression curve was used to calculate the concentration of each sample.

### 2.10. Cytokine Measurement

Serum levels of IL-1, IL-6, IL-10, and TNF-*α* were measured using ELISA (Shanghai Enzymology Laboratory Equipment Co. Ltd., Shanghai, China), according to the manufacturer's instructions on the kit. The standard concentration was set as the abscissa, and the OD value was set as the ordinate; a standard curve was then drawn, and the concentration of corresponding cytokines was calculated.

### 2.11. Statistical Analysis

All results are presented as mean ± SD. Results were analyzed using GraphPad Prism version 5.0 program. One-way analysis of variance was used for comparison between groups. *p* < 0.05 was considered statistically significant.

## 3. Results

### 3.1. *Glycyrrhiza* Polysaccharide (GPS) Preparation

GPS was obtained via water extraction and alcohol precipitation, and the yield of GPS was 7.35 ± 0.12% (*n* = 3).

### 3.2. Total GPS Sugar and Protein Content

Total sugar content was assayed by the phenol-sulfate method, with glucose as the standard. Average GPS polysaccharide content was 43.50 ± 1.97% (*n* = 5). Total protein content was measured by the Coomassie brilliant blue method. Average GPS protein content was 4.50 ± 0.12% (*n* = 5).

### 3.3. GPS Molecular Weight

GPS's molecular weight was determined via high-performance gel permeation chromatography. The standard molecular weight curves were based on dextran of differing molecular weights: *y* = 25.749*x* + 0.0572 (*R*^2^ = 0.9949). Average molecular weight of GPS was determined to be 1.26 × 10^5^ Da.

### 3.4. GPS Monosaccharide Composition

Based on retention times, GPS's monosaccharide composition was determined to be rhamnose, glucuronic acid, galacturonic acid, glucose, and galactose; the relative content ratio of each monosaccharide was 1.6 : 2.0 : 4.3 : 1 : 26, respectively, according to peak area.

### 3.5. General Mice Condition

Mice in the normal control group (NC) performed well and exhibited a normal mental status. After 6 days of DSS treatment, the mice in the model group (Model) had sluggish eyes, laid down, slumped, consumed significantly reduced water and food intake, and had loose or bloody stool. After 8 days of therapeutic administration, with GPS or SASP, the condition of the mice improved.

### 3.6. GPS Decreases DAI Scores of UC Mice

**Figure 1:**
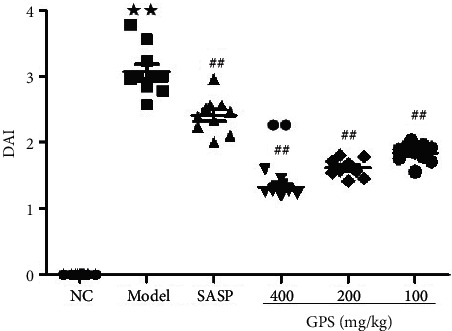
Effects of GPS on DAI scores in UC mice (*n* = 10). ^★★^*p* < 0.01 vs. normal control; ^##^*p* < 0.01 vs. model; ^●●^*p* < 0.01 vs. SASP. NC: normal group; SASP: positive drug group; Model: model group; GPS: *Glycyrrhiza* polysaccharide group.

Animals' DAI scores are shown in Figure 1. Compared with the NC by Wilcoxon rank-sum test, the DAI score of the Model (3.073 ± 0.362) was increased. Compared with the Model, the DAI scores of GPS (GPSH, 1.326 ± 0.119; GPSM, 1.168 ± 0.131; GPSL, 1.841 ± 0.141) and SASP groups (2.413 ± 0.268) were significantly decreased. Furthermore, the DAI score of the high-dose GPS group was significantly reduced compared with the SASP group, indicating that GPS has a superior UC relief effect and in a dose-dependent manner.

### 3.7. GPS Inhibits DSS-Induced Weight Loss in Mice

**Figure 2:**
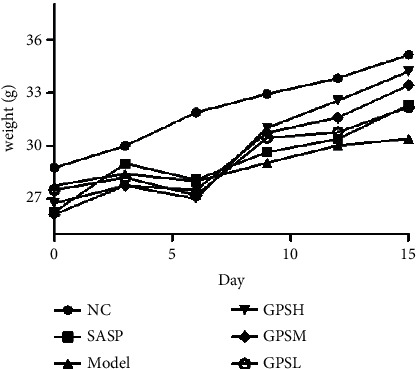
Effects of GPS on body weight changes in UC mice (*n* = 10). NC: normal control group; SASP: positive drug group; Model: model group; GPSH: high dose of GPS group; GPSM: middle dose of GPS group; GPSL: low dose of GPS group.

In the NC group, body weight increased slightly during the entire observation period. After 3 days of DSS administration, mice began to exhibit a weight loss trend (Figure 2). When treated with GPS or SASP, mice weights tended to increase from day 7 to day 15 and their mental statuses notably improved. On day 11, the GPSHs' body weights (34.21 ± 2.13) had returned to near those of the NC (35.16 ± 2.03). These results indicate that GPS can inhibit DSS-induced mice weight loss.

### 3.8. GPS Colon Length Effect

**Figure 3:**
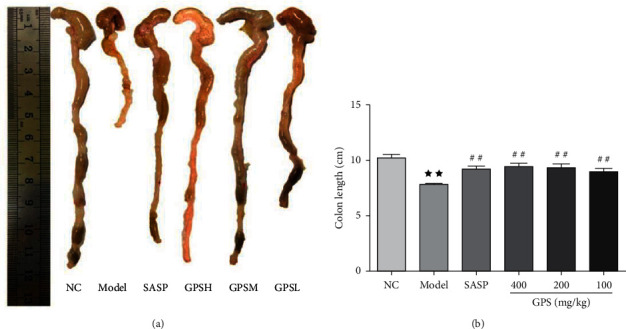
Effects of GPS on colon length of UC mice (*n* = 10). ^★★^*p* < 0.01 vs. normal control; ^##^*p* < 0.01 vs. model. NC: normal group; SASP: positive drug group; Model: model group; GPS: *Glycyrrhiza* polysaccharide group.

In UC Model mice (8.25 ± 0.54), the length of the colon was significantly shortened compared with the NC (10.38 ± 0.96). Both GPS (GPSH, 9.76 ± 0.65; GPSM, 9.69 ± 0.72; GPSL, 9.68 ± 0.93), and SASP (9.65 ± 0.38) treatments alleviated the effects of DSS on colon shortening and were significantly different from the Model (Figure 3).

### 3.9. GPS Effect on UC Mice Spleen Weight

**Figure 4:**
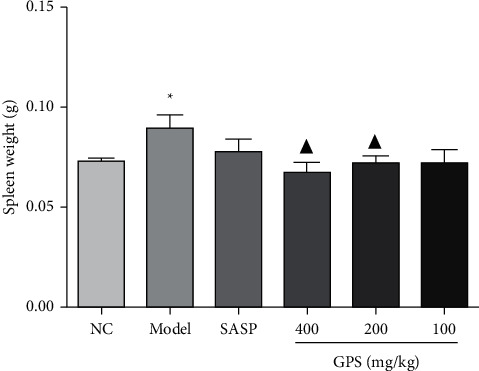
Effects of GPS on spleen weight in UC mice (*n* = 10). ^*∗*^*p*< 0.05 vs. normal control; ^▲^*p* < 0.05 vs. Model. NC: normal group; SASP: positive drug group; Model: model group; GPS: *Glycyrrhiza* polysaccharide group.

Compared with the NC (0.073 ± 0.005), the spleen weight of UC Model mice (0.089 ± 0.020) were increased significantly. After GPS treatment (GPSH, 0.067 ± 0.015; GPSM, 0.072 ± 0.010; GPSL, 0.072 ± 0.019), spleen weight significantly decreased compared with the Model (Figure 4). SAPS (0.078 ± 0.020) treatment also decreased spleen weight compared to the Model; however, the difference was not statistically significant. These findings suggest that GPS can ameliorate splenomegaly in UC mice.

### 3.10. Histological Examination

**Figure 5:**
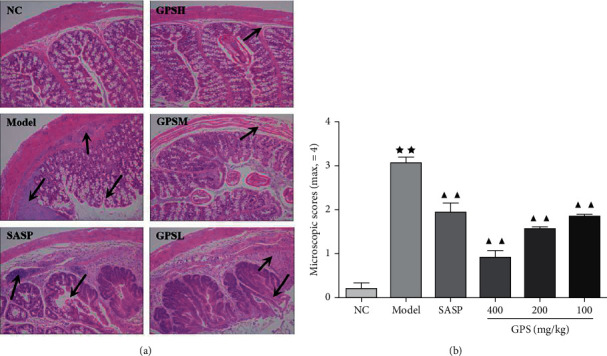
GPS treatment ameliorated mucosal damage of UC mice. (a) Microscopic images of the colon (×100). (b) Histopathological score of mucosal damage in UC mice treated with GPS (*n* = 7). ^*∗∗*^*p* < 0.01 vs. NC; ^▲▲^*p* < 0.01 vs. Model. NC: normal group; SASP: positive drug group; Model: model group; GPS: *Glycyrrhiza* polysaccharide group.

No histopathological changes were observed in the NC. In contrast, the Model mucosa exhibited mixed inflammatory infiltrates and mucosal damage (black arrows), epithelial cell shedding, and tissue hyperplasia (Figure 5(a)). Compared to the Model group, histopathological changes were significantly ameliorated in GPS and SASP groups, as demonstrated by histopathological score (Figure 5(b)).

### 3.11. GPS Effect on FITC-Dextran Concentration in DSS-Induced Acute UC Mice

FITC-dextran has a molecular weight of about 40 kDa; due to this high molecular weight, it does not easily diffuse into the blood via the intestinal epithelium, and so FITC-dextran serum concentration in serum can effectively serve as a fluorescent agent reflecting intestinal permeability [21]. UC is universally accompanied by an increase in intestinal inflammation and an increase in intestinal permeability [3].

**Figure 6:**
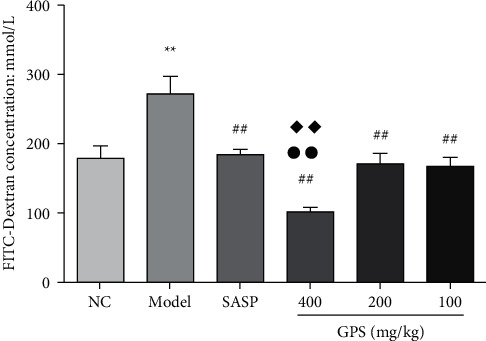
Effects of GPS on FITC-dextran concentration in UC mice (*n* = 10). ^◆◆^*p* < 0.01, ^*∗∗*^*p* < 0.01 vs. normal control; ^##^*p* < 0.01 vs. Model; ^●●^*p* < 0.01 vs. SASP. NC: normal group; SASP: positive drug group; Model: model group; GPS: *Glycyrrhiza* polysaccharide group.

Compared with the NC (180 ± 57), serum FITC-dextran content in the Model (271 ± 94) increased significantly; compared with the Model, serum FITC-dextran content was significantly decreased in the GPS and SASP (185 ± 24) groups. Notably, the serum FITC-dextran content of the GPSH (102 ± 17) was significantly decreased compared to the SASP and even significantly below that in the NC (Figure 6). These results demonstrate that GPS treatment can significantly reduce the increase in intestinal permeability in UC mouse model induced by DSS.

### 3.12. Effects of GPS on D-Lactic Acid (D-LAC) Concentration in DSS-Induced UC Mice

D-lactic acid is a metabolite produced by intestinal bacterial fermentation, entering blood circulation when intestinal permeability increases. Accordingly, serum lactic acid content can be used as an additional indicator of intestinal permeability [22].

**Figure 7:**
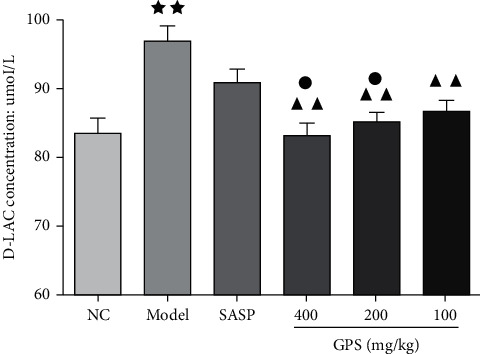
Effects of GPS on D-LAC concentration in UC mice (*n* = 10). *p* < 0.01 vs. normal control; ^▲▲^*p* < 0.01 vs. Model; ^●^*p* < 0.05 vs. SASP. NC: normal group; SASP: positive drug group; Model: model group; GPS: *Glycyrrhiza* polysaccharide group.

Compared with the NC (83.41 ± 6.54), serum D-lactic acid content of the Model (96.84 ± 6.66) were significantly increased. Compared with the Model, the serum D-lactic acid content in the GPS treatment groups decreased significantly. Furthermore, the serum D-lactic acid content of the GPSH (83.17 ± 5.22) and GPSM (85.07 ± 4.09) were significantly lower than the SASP group (90.82 ± 5.89) (Figure 7). This experiment demonstrates that GPS treatment can significantly reduce the increase in intestinal permeability associated with DSS-induced UC, at both high and medium doses.

### 3.13. GPS Effect on Cytokine Levels in DSS-Induced UC Mice

**Figure 8:**
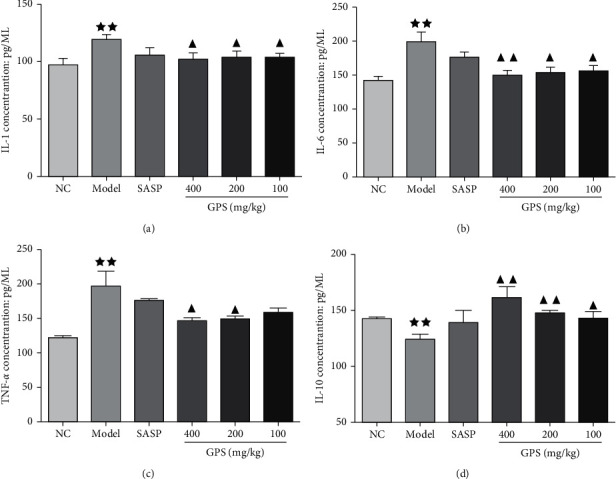
Effects of GPS on the levels of IL-1 (a), IL-6 (b), TNF-*α* (c), and IL-10 (d) in UC mice (*n* = 10). ^★★^*p* < 0.01 vs normal control; ^▲^*p* < 0.05; ^▲▲^*p* < 0.01 vs. model. NC: normal group; SASP: positive drug group; Model: model group; GPS: *Glycyrrhiza* polysaccharide group.

TNF-*α*, IL-1, IL-6, and IL-10 are important cytokines involved in immune responses and mediate inflammatory responses [23]. Compared with the NC, the serum levels of IL-1, IL-6, and TNF-*α* in the Model were significantly increased and the serum IL-10 levels were significantly reduced. Compared with the Model, serum levels of TNF-*α*, IL-1, and IL-6 in the GPS mice groups at each dose were significantly reduced, and serum IL-10 levels significantly increased (Figure 8). These results suggest that GPS treatment can inhibit the inflammatory response in UC mice.

## 4. Discussion

This study demonstrates that GPS can relieve the weight loss, colon shortening, diarrhea, and stool bleeding clinical symptoms associated with DSS-induced UC mice. Furthermore, GPS inhibits the release of proinflammatory factors IL1, IL6, and TNF-*α,* and promotes the production of anti-inflammatory factor IL-10, thereby inhibiting the inflammatory response. The multiple indicators observed in this study exhibit that GPS's therapeutic effect on DSS-induced UC mice is superior to the clinical drug SASP. GPS exhibited statistically superior performance in DAI score and intestinal permeability, and nonsignificant enhancements over SASP in the other tested indicators such as spleen weight, proinflammatory factors IL1, IL6, and TNF-*α* and anti-inflammatory factor IL-10. Given its excellent safety profile of polysaccharides [24], as significant weakness in current UC therapies, this study highlights GPS`s remarkable potential as a candidate for the treatment of irritable bowel syndrome.

Although GPS can effectively reduce intestinal permeability and intestinal inflammation, its mechanism remains unclear. One of the primary characteristics of UC is the increase in intestinal permeability [3], which allows antigens in the digestive tract enter into the body and induce inflammation; inflammation can lead to mucosal damage and aggravate UC symptoms. A reduction in intestinal permeability could inhibit the entry of macromolecular antigens into the body, subsequently reducing the inflammatory response and therefore mucosal damage. In other words, both increased inflammatory response and increased intestinal permeability are likely causes of UC, and the two are mutually causal. This study shows that GPS can reduce both intestinal permeability and the inflammatory response but does not elucidate which effects occur first. According to the serum FITC-dextran content, the intestinal permeability of FITC-dextran in the GPS treatment groups were even lower than that in the NC; the difference between the NC and GPSH was statistically significant, with the NC expressing no UC symptoms and normal proinflammatory factors. This result is consistent with the observation that GPS significantly improved the expression of ZO-1 in an immunosuppressed mice model over even the control group (attachment materials). Therefore, we hypothesize that GPS reduces intestinal permeability prior to inducing anti-inflammatory effect and that GPS's mechanism in alleviating DSS-induced UC may be related to its promotion of intestinal mucosal barrier function. Follow-up research will focus on possible molecular mechanisms by which GPS affects intestinal permeability.

The intestinal mucosal barrier is a crucial barrier in the body against external pathogens and toxins. If the intestinal mucosa is damaged under various pathological effects, it will cause a variety of inflammatory diseases, notably ulcerative colitis, infectious colitis, and parenteral diseases [25]. The restoring or improving the intestinal mucosal barrier plays an important role in curing these diseases and is a potential therapeutic strategy for ulcerative colitis; many drugs such as bilirubin, chitosan, and fucoidan may alleviate enteritis symptoms via enhancing intestinal barrier function.

The traditional licorice usage has shown an antiulcer effect, an effect which may be related to mucosal repair. GPS's effect on the reduction of intestinal permeability could indicate that licorice has a mucosal repair effect. Previous studies have shown that glycyrrhizin and glycyrrhetinic acid from licorice have antiulcer effects and attributed these to their anti-inflammatory effects as well as identified them as the primary active compounds [23]. This paper challenges these assertions, highlighting GPS as—at least—another active component of licorice, partially responsible for licorices intestinal permeability and anti-inflammatory effects.

Polysaccharides are nutrients of intestinal bacteria, which affect gut microbiota composition [26]. Changes in the gut microbiota have been linked to inflammation responses, in both positive and negative angles. Further, fecal microbiota transplantation has been shown relieve the symptoms of UC or irritable bowel disease [14]. Some studies have found that GPS has a regulatory effect on intestinal microflora, highlighting another potential mechanism of action [13]. Whether GPS's therapeutic effect on UC is related to changes in gut microbiota is another question warranting further exploration.

## 5. Conclusion

GPS can relieve the clinical symptoms of DSS-induced UC mice as well as regulate inflammatory factors, inhibiting the inflammatory response. Highlighting an understudied active compound in millennia-old licorice medication, this study provides an experimental basis for the use of GPS in UC treatment and indicates intestinal permeability to be its primary mechanism of action.

## Figures and Tables

**Table 1:** The DAI scoring criteria.

DAI score	Weight loss (%)	Stool consistency	Occult/gross bleeding
0	None	Normal	Normal
1	1–5	Loose stools	Hemoccult positive
2	5–10		
3	10–20		
4	>20	Diarrhea	Gross bleeding

## Data Availability

The data used and analyzed during the current study are available from the first author and corresponding author on reasonable request.

## Supplementary Materials

Supplementary Materials Figure S1: effects of GPS and DGPS on tight junction protein ZO-1 expression in immunosuppressed mice (‾*x* ± *s*, *n* = 3). ^△^*p* < 0.05; ^△△^*p* < 0.01 vs. normal control; ^*∗*^*p* < 0.05, ^*∗∗*^*p* < 0.01 vs. model; ^##^*p* < 0.01 vs. GPS. NC: normal group; PC: positive drug group; Model: model group; DGPS: *Glycyrrhiza* polysaccharide hydrohydrolytic group; GPS: *Glycyrrhiza* polysaccharide group.
