# Supplementary material for: Glycyrrhiza Polysaccharide Alleviates Dextran Sulfate Sodium-Induced Ulcerative Colitis in Mice
Source: Evid Based Complement Alternat Med. 2022 Apr 8;2022:1345852. doi: 10.1155/2022/1345852 (PMC9012628; doi:10.1155/2022/1345852)
Supplement: Supplementary Materials — Figure S1: effects of GPS and DGPS on tight junction protein ZO-1 expression in immunosuppressed mice (‾x ± s, n = 3). △p < 0.05; △△p < 0.01 vs. normal control; ∗p < 0.05, ∗∗p < 0.01 vs. model; ##p < 0.01 vs. GPS. NC: normal group; PC: positive drug group; Model: model group; DGPS: Glycyrrhiza polysaccharide hydrohydrolytic group; GPS: Glycyrrhiza polysaccharide group. [file 1345852.f1.doc]

Effects of GPS on expression of small intestine tight junction protein ZO-1 in immunosuppressed mice induced by cyclophosphamide. After being induced by cyclophosphamide, the expression of ZO-1 in mice was significantly decreased and the mechanical barrier of intestinal mucosa was significantly damaged. Compared with the NC group, the expression of ZO-1 in the Model group was significantly decreased (*P<0.01*). The expression of ZO-1 was significantly increased (*P<0.01*) after treat with positive drug and GPS, and the expression of Zo-1 increased in a dose-dependent manner.


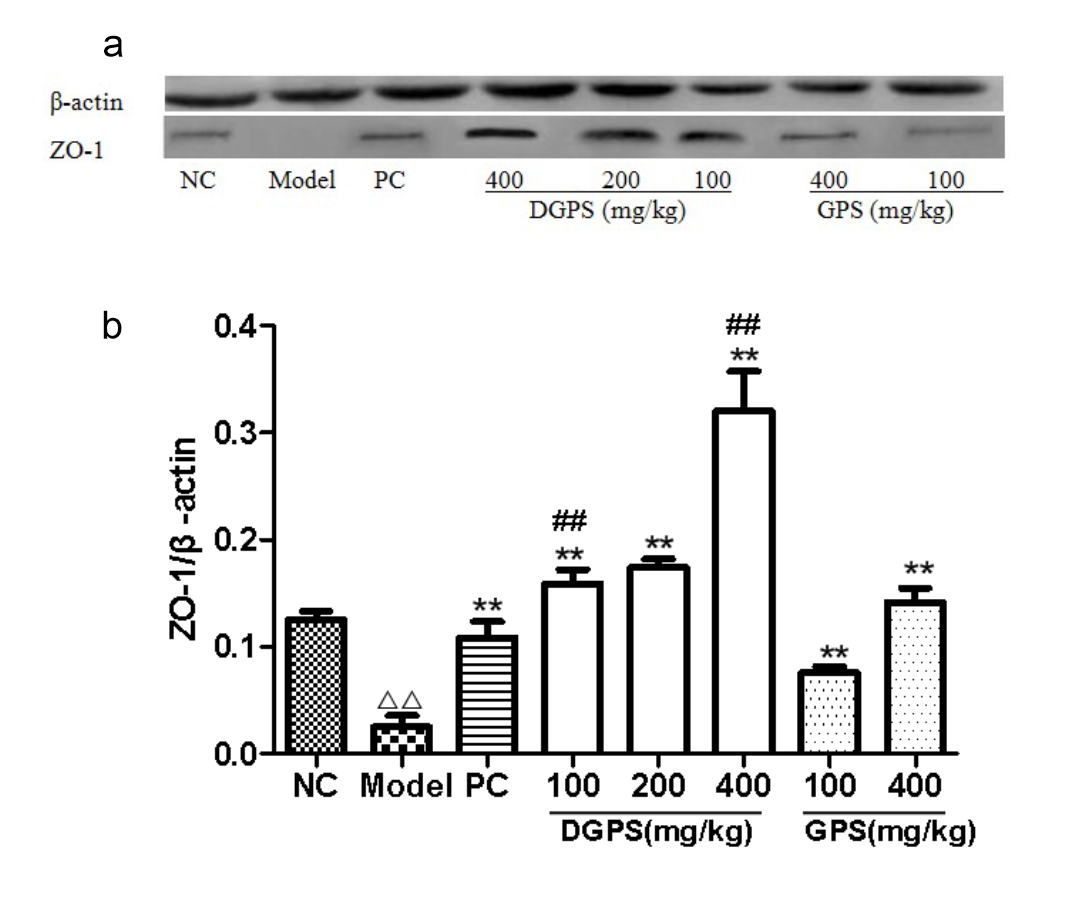


Figure S1: Effects of GPS and DGPS on tight junction protein ZO-1 expression in immunosuppressed mice (*x*±s, *n*=3 )**.** Note：^△^*p*<0.05,^△△^*p*<0.01 vs. normal control; **p*<0.05, ***p*<0.01 vs. model ;^##^*p*<0.01vs.GPS. NC: normal group; PC: positive drug group; Model: model group; DGPS: glycyrrhiza polysaccharide hydrohydrolytic group; GPS: glycyrrhiza polysaccharide group.
